# Breast lump as the initial presentation of metastatic uterine leiomyosarcoma

**DOI:** 10.3325/cmj.2025.66.305

**Published:** 2025-08

**Authors:** Ilker Sengul, Demet Sengul

**Affiliations:** 1 Division of Endocrine Surgery, Department of General Surgery, Giresun University Faculty of Medicine, Giresun, Turkey; *ilker.sengul.52@gmail.com*; 2 Department of Pathology, Giresun University Faculty of Medicine, Giresun, Turkey

To the Editor:

We read with interest the article “*Breast Lump as the Initial Presentation of Metastatic Uterine Leiomyosarcoma: a Case Report and Comprehensive Literature Review*” (1) published in 2024 in the *Croatian Medical Journal*. Ibisevic et al (1) highlight a rare and important clinical presentation of uterine leiomyosarcoma (uLMS), where a breast mass served as an initial indication of metastatic disease. The authors provide a valuable summary of this uncommon metastatic pattern. While the article effectively documents this rare occurrence, the authors underscore a significant diagnostic challenge illustrated by the case: the initial misclassification of the primary uterine tumor. According to the report, the largest uterine nodule (200 × 130 mm) was initially diagnosed in 2017 as an atypical (symplastic) leiomyoma following total abdominal hysterectomy. Based on these findings, routine gynecologic care was recommended. However, five years later, after the patient presented with a breast mass subsequently diagnosed as metastatic leiomyosarcoma, a re-review of the original uterine samples from 2017 revealed that the largest nodule exhibited clear morphologic features of uLMS, including severe pleomorphism/atypia, 12/10 hpf mitoses, and tumor cell necrosis. The sources explicitly state that microscopic tumor cell necrosis and more than ten mitoses/hpf indicate uLMS, not symplastic (atypical) leiomyoma. This initial misdiagnosis meant that an aggressive cancer with high metastatic potential might not have been identified and treated appropriately in 2017. Actually, the delay in diagnosis until metastatic disease became evident in multiple sites (the lungs, liver, L3 vertebra) highlights the potential consequences of missing key diagnostic criteria or challenges in applying them consistently. The authors themselves acknowledge the difficulty in distinguishing uLMS from leiomyoma, including symplastic leiomyoma, stating that while clear diagnostic criteria exist, in some cases, the distinction can be challenging. Their case serves as a stark illustration of the real-world impact of overlooking these criteria or an ambiguous initial presentation. It reinforces the authors' conclusion that a comprehensive diagnostic work-up, including re-evaluation of previous pathology and interdisciplinary collaboration, is crucial for accurate diagnosis and appropriate treatment selection. While detailing a rare metastatic site, this case report serves as a reminder of the persistent challenges in the accurate initial diagnosis of uterine leiomyosarcoma, particularly its distinction from benign or atypical variants. It highlights the importance of applying rigorous histopathological criteria and considering the possibility of malignancy in suspicious uterine lesions, even those initially thought to be benign or atypical. This issue merits further investigation (2). We thank Ibisevic et al (1) for their valuable study on a breast lump as the initial presentation of metastatic uterine leiomyosarcoma. *Bene diagnoscitur*, *bene curatur.*
